# Assessing the role of imported cases on the establishment of SARS-CoV-2 Delta variant of concern in Bolton, UK

**DOI:** 10.1017/S0950268821002776

**Published:** 2022-05-12

**Authors:** Joseph Shingleton, Thomas Finnie, Nick Gent, Emma Bennett

**Affiliations:** Public Health Guidance and Expertise Joint Modelling Cell, Public Health England, Salisbury, UK

**Keywords:** Covid-19, Mathematical Modelling, Infectious Disease

## Abstract

This paper presents a method used to rapidly assess the incursion and the establishment of community transmission of suspected SARS-CoV-2 variant of concern Delta (lineage B.1.617.2) into the UK in April and May 2021. The method described is independent of any genetically sequenced data, and so avoids the inherent lag times involved in sequencing of cases. We show that, between 1 April and 12 May 2021, there was a strong correlation between local authorities with high numbers of imported positive cases from India and high COVID-19 case rates, and that this relationship holds as we look at finer geographic detail. Further, we also show that Bolton was an outlier in the relationship, having the highest COVID-19 case rates despite relatively few importations. We use an artificial neural network trained on demographic data, to show that observed importations in Bolton were consistent with similar areas. Finally, using an SEIR transmission model, we show that imported positive cases were a contributing factor to persistent transmission in a number of local authorities, however they could not account for increased case rates observed in Bolton. As such, the outbreak of Delta variant in Bolton was likely not a result of direct importation from overseas, but rather secondary transmission from other regions within the UK.

## Introduction

Cases of COVID-19 in the UK saw a steady decline during the first part of 2021 [1], thanks to a combination of an effective vaccination programme and strict non-pharmaceutical interventions [2]. During this period, the primary strain of SARS-CoV-2 in the UK was B.1.1.7, or Alpha variant – making up between 75% and 95% of all new cases [3]. While cases were declining at a national scale, there was a degree of local heterogeneity in this decline. A number of Lower Tier Local Authorities (LTLA), particularly those in urban centres, saw persistent community transmission and a considerably slower decline in case numbers [4].

The situation was quite different in India, however, with a rapid increase in confirmed cases of COVID-19 during March and April 2021 [5]. This surge in cases was driven primarily by B.1.617.2, or Delta variant [5]. Concerns over the threat of incursion of the Delta variant into the UK led to the temporary suspension of all non-essential inbound travel from India on 23 April 2021 [6].

**Fig. 1.**
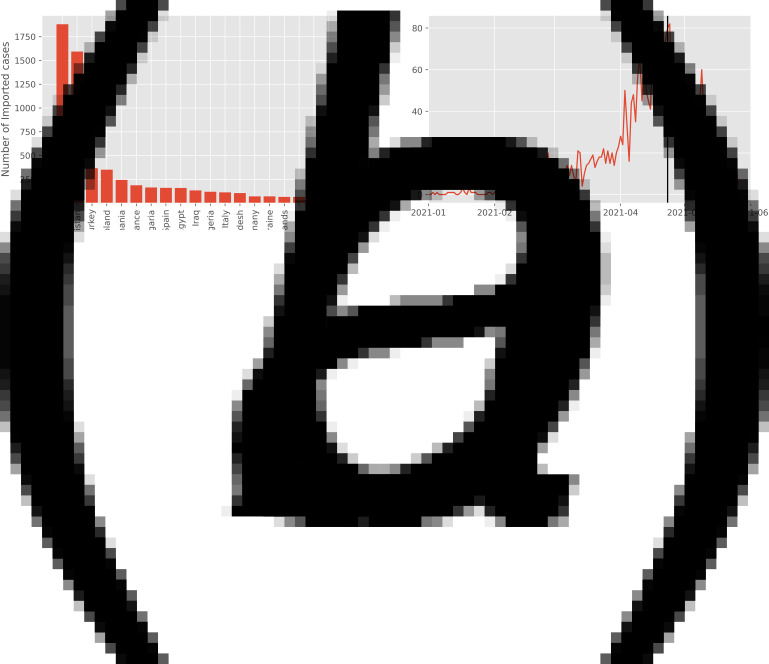
(a) The number of imported positive cases from the top 20 most frequent countries of origin between 1 March and 12 April 2021 and (b) timeseries of importations from India since 1 January 2021. The black line indicates the date on which all non-essential inbound travel from India was suspended (23 April).

Testing of inbound travellers to the UK, conducted within the first 48 h of arrival and repeated 8 days after arrival, shows that importations from India accounted for 26.7% of all imported positive cases between 1 March and 23 April, the highest rate of any country (Fig. 1a). While importation numbers did fall quickly after the implementation of tighter border restrictions (Fig. 1b), the volume of imported cases from India over the period meant that an incursion of the Delta variant was likely.

During the second part of April, Bolton, an LTLA in the north west of England, saw a sharp resurgence in cases. Further, a large proportion of these cases were in Indian and Pakistani communities [7]. Genetic sequencing of cases over this period confirmed that the epidemic was driven predominantly by the Delta variant.

Genetic sequencing techniques have played a vital role in understanding the threat posed by imported cases. However, despite the UK having the shortest delay between case confirmation and sequencing, with a median lag time of 16 days [8], it remains a slow and unresponsive data stream. As such, genomic sequencing is often unsuitable for providing the rapid analyses required to respond to newly emerging outbreaks.

The situation in India, along with the new incursion of Delta variant in Bolton, meant that rapid assessment of the origin and destination of imported positive cases was crucial. In this paper, we show that analysis of the inbound traveller testing data was able to provide evidence that LTLA COVID-19 infection rates over the 6-week period between 1 March and 12 April 2021 were geographically correlated with rates of importation of positive cases from India over the period. Further, we show that Bolton was an outlier in this relationship – having very high case rates despite relatively few importations. Finally, using an SEIR transmission model, we show that the importation of positive cases was an important contributing factor to the persistent transmission observed in a number of LTLAs; however, it was not sufficient in explaining the ongoing transmission observed in Bolton.

## Methods

### Identifying the relationship between imported cases and case rates

Since 15 February 2021, the UK has implemented a policy of testing all inbound travellers for COVID-19 within 48 h of arrival to the UK, and then again 8 days after arrival. Data regarding the travellers' origin country, and destination postcode are also recorded. The analysis presented in this paper aims to explore the relationship between importation rates and COVID-19 case rates for LTLA in England.

We are primarily concerned with COVID-19 importations from India; however, we also compare these with the aggregated importations from all countries. Importations are limited to those occurring over the 6-week period between 1 March and 12 May 2021. We account for population effects by considering each LTLA's importation rate – calculated as the number of importations per 10 000 population.

COVID-19 case rates in each LTLA are calculated from confirmed cases [9], and are again given as a rate per 10 000 population. We only consider cases which were initially identified through polymerase chain reaction (PCR) testing; rather than either lateral flow device (LFD) testing, or LFD testing with confirmatory PCR. This somewhat mitigates the effects of surge testing, as well as testing within schools and businesses.

We also provide the analysis on a finer geographic scale by investigating the importation rate and case rate per 1000 population of each Middle Super Output Area (MSOA) in nine LTLAs. These LTLAs have been selected due to having either >30 cases per 10 000 population and non-zero imported positive cases, or with >2 imported positive cases per 10 000 population.

### Validating importation rates

A reasonable concern one might have with this analysis is that it offers no way of knowing whether the observed importation rates to Bolton were accurate. To address this, we must show that Bolton had as many importations as we might expect, given its economic, demographic and geographic characteristics.

To do this, we use an artificial neural network trained on demographic data at MSOA level. The model uses 161 explanatory variables, covering age distributions, countries of birth, economic activity, ethnicity, household structures, living arrangements, socio-economic classification and religion; all taken from 2011 census data. This was supplemented with more recent data at LTLA level covering 2019 estimates for religion and ethnicity. Geographic location was also included in the model, encoded as the latitude and longitude of each MSOA's population weighted centroid. All variables were scaled such that they have mean of zero and standard deviation of one before being passed into the model. We use Principle Component Analysis to reduce the dimensionality of the model from 161 to 35 explanatory variables, while retaining 95% of the data's variance.

The data were split into training and testing sets, with 79.5% of LTLAs (252/317) being used for training data. Bolton was placed in the testing set to ensure reliable validation, otherwise LTLAs were randomly allocated as testing or training data. The 252 LTLAs making up the training set comprised of 5517 individual MSOAs, with the testing set consisting of a further 1274 MSOAs, giving a true training split of 81.2%. A further 20% of the training data (1104 MSOAs) were used to validate the model during training, helping to avoid model overfitting.

The model architecture is described in Section S1 in the Supplementary Material. The model was built in Python (v3.9) using the Keras (v2.3.1) machine learning package. The results of the Neural Network model were checked against a second analysis using a Gradient Boosting Regression model trained on identical input data. The Gradient Boosting Regression model was built using the Sci-Kit Learn package (v0.23.1) for Python. Details of this model are also given in the Supplementary Materials.

### Transmission modelling

The method described in section ‘Identifying the relationship between imported cases and case rates’ provides a quick and effective way of identifying the relationship between imported cases and COVID-19 case rates. However, it does not provide any indication of whether importations were sufficient in explaining the ongoing transmission seen in a number of LTLAs. In order to better understand this we must demonstrate a causative link between importations and case rates.

We employ an SEIR transmission model, fitted to LTLA case data, to identify this causative link. The model, described in equations 1–8, uses multiple compartments for the exposed population (*E*_0_, *E*_1_, *E*_2_), simulating the Erlang distributed incubation periods observed in the literature [10, 11]. Further, we separate the infectious (*I*_0_) compartments into those cases that are detected by COVID-19 surveillance streams (*I*_*d*_) and those that remain undetected (*I*_*u*_) [11]. We assume that detected infectious individuals undergo self-quarantine, reducing their transmission rate by a factor *ν* [12]. The Removed compartment (*R*) comprises of recovered individuals and fatalities.

The vaccination programme is modelled as taking individuals from the susceptible (*S*) to the removed (*R*) compartments at a constant rate, calculated as the mean daily vaccinations over the period. This assumption agrees with observations which suggest that the vaccination rate in the UK was broadly constant between January and May 2021 [1]. We have made no attempt to account for differences between first and second doses of the vaccine, and assume all vaccines are 80% effective [13–15].

The model is described by the following system of Ordinary Differential Equations:1
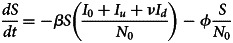
2
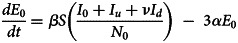
3
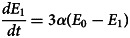
4
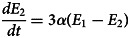
5
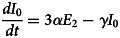
6
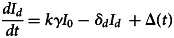
7
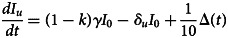
8
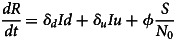


We use Approximate Bayesian Computation (ABC) to parameterise the model, based on observations of case numbers in each LTLA between 10 January and 1 April 2021. The fitting process uses a negative binomial loss function – a common choice when dealing with over-dispersed epidemiological data [16, 17]. A list of the model parameters and initial values is provided in Tables S1 and S2 in the Supplementary Materials. The ABC fitting process provides 1000 posterior parameterisations for each LTLA. Model development and ABC fitting was completed using the PyGOM package for Python.

All model parameters are fitted using ABC, with the exception of vaccine efficacy *ϕ* = 0.8 [13–15], self-isolation rate *ν* = 0.1 [12] and latency rate *α* = 0.196 days-1 [10, 11]. The initial values of each state are also identified via ABC, except for *I*_*d*_(0) = *T*_*c*_*y*(0); where *T*_*c*_ is the mean generation time for COVID-19, taken to be *T*_*c*_ = 9 days [18], and *y*(0) is the first value in the case data. Sensitivity of the results across *ϕ* ∈ {0.4, 0.6, 0.9}, *ν* ∈ {0.05, 0.2, 0.5} and *T*_*c*_ ∈ {3, 6, 12, 15} is shown in Tables S1–S3 in the Supplementary Materials.

The model is run twice for each LTLA. The first run uses the training data to estimate the continued trajectory of cases in the absence of any imported cases. In the second run, we introduce imported positive cases to the model after the fitting period. In order to model cases which are not captured by border screening, estimated to be around 10% of cases [19], we introduce imported cases to both the *I*_*d*_ and *I*_*u*_compartments. On day *t*, we introduce Δ(*t*) cases to *I*_*d*_, and a further 0.1Δ(*t*) to *I*_*u*_.

We use two metrics to assess the accuracy of the projections. First, we assess the broad accuracy by considering the root mean squared error (RMSE) between the log-transformed observed and predicted cases. Secondly, we also consider how well the model captures the observed growth rate in cases after the fit period. Growth rates are obtained by fitting a generalised linear model with a negative binomial link function to the log-transformed case data and projections – the coefficient of this model gives us an estimate for growth rate.

## Results

### Imported cases and case rates

**Fig. 2.**
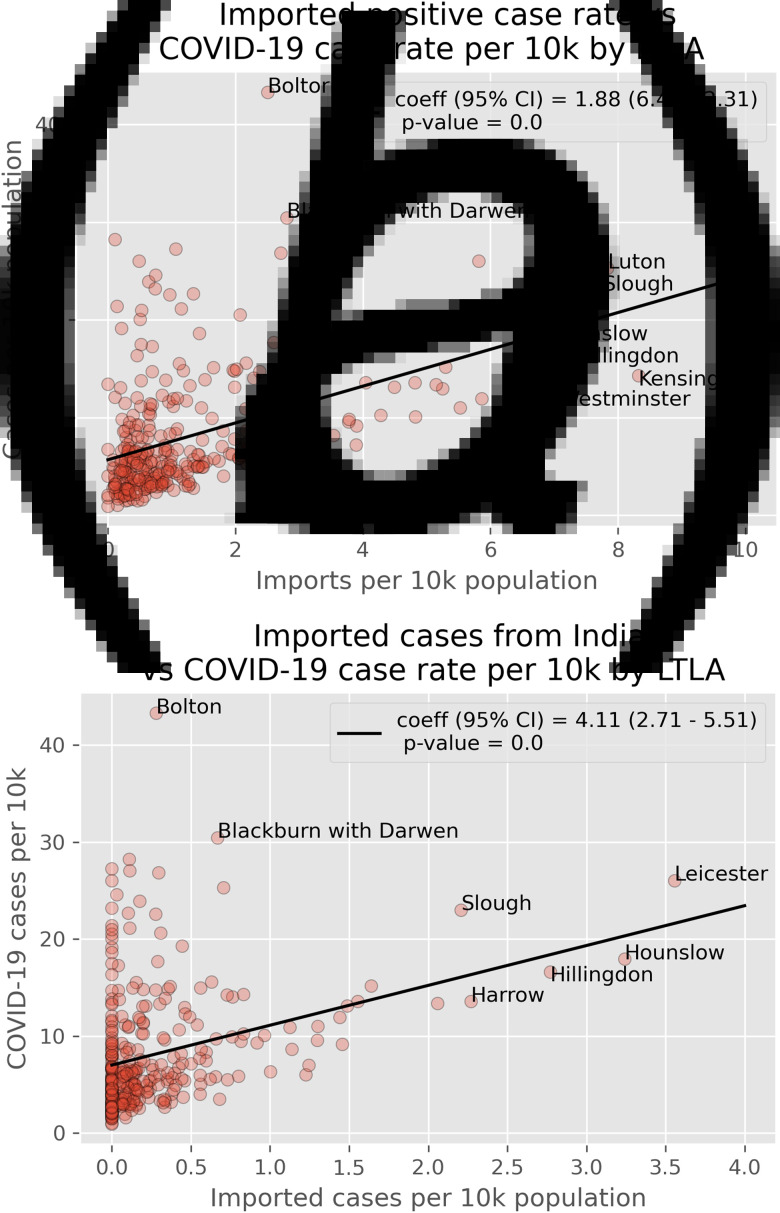
Plots showing (a) imported cases from all countries *vs*. COVID-19 case rates and (b) imported cases form india *vs*. COVID-19 case rates. All importation and case rates are calculated over the 6-week period between 01/04/21 and 12/05/21.

Figure 2 shows the relationship between case rates and importations from (a) all countries and (b) India between 1 April and 12 May 2021. Both plots indicate a positive relationship between the number of imports to an LTLA and the case rate. This relationship is stronger when considering only importations from India. However, there remain a number of LTLAs which saw high case rates despite limited importations. In particular, Bolton saw the highest case rates of any LTLA, despite importations rates of only 0.28 imports per 10 k – only slightly higher than the mean rate of 0.25 imports per 10 k.

### Imported cases and local case rates

To gain a better understanding of the relationship between imported cases from India and COVID-19 case rates, it is necessary to consider local scale effects. In this section, we consider the relationship between importations and MSOA case rates in nine LTLAs: Blackburn with Darwen, Bolton, Ealing, Harrow, Hillingdon, Hounslow, Kirklees, Leicester and Slough. The selection criteria for these LTLA were case rates of >30 cases per 10 k with non-zero importations (Blackburn with Darwen, Bolton, Kirklees), or Indian importation rates >2 importations per 10 k (Ealing, Harrow, Hillingdon, Hounslow, Leicester and Slough).

**Fig. 3.**
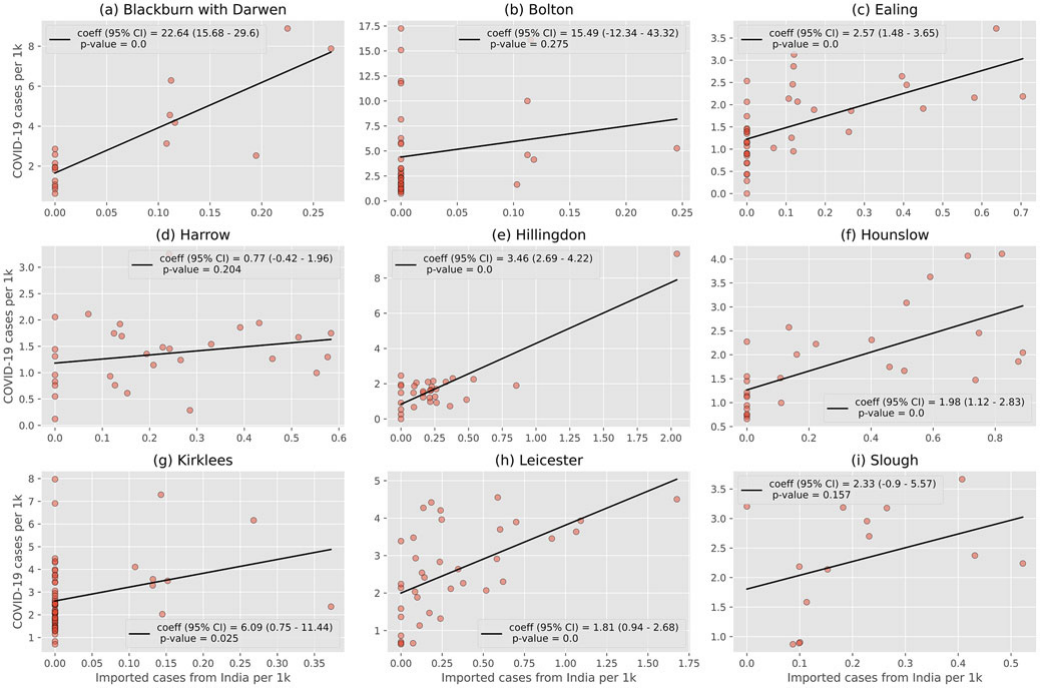
Relationship between importation rate of positive cases from India per 1 k population and COVID-19 case rate per 1 k population in MSOA across nine LTLA. A statistically significant relationship (*P* < 0.001) is observed for Blackburn with Darwen, Ealing, Hillingdon, Hounslow and Leicester. The results for Bolton are amongst the least statistically significant.

This local-level relationship is shown in Figure 3. Blackburn, Ealing, Hillingdon, Hounslow and Leicester all had high case rates in MSOA with high Indian case importation rates (*P* < 0.01), indicating that these LTLA often saw outbreaks in areas with high importations. No correlation was identified for Bolton, Kirklees or Harrow. Note, however, that the result for Hillingdon is driven primarily by a single MSOA with high importations and high case rates. When this MSOA is removed from the analysis the relationship no longer holds (*P* = 0.124). Similar analyses investigating imported cases from both Pakistan and all countries are discussed in the Supplementary Material.

### Verifying importation rates

**Fig. 4.**
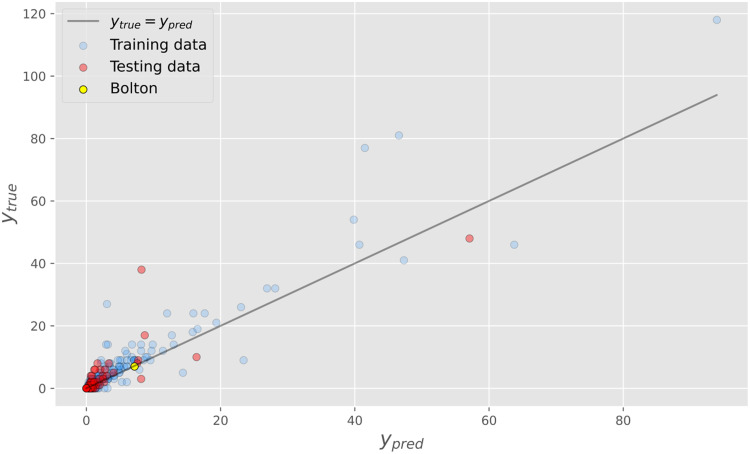
Observed (*y*_*true*_) and predicted (*y*_*pred*_) importations from India for each LTLA in England. The predictions are made using a deep neural network trained on MSOA demographic data. There is no indication that the observed importations in Bolton were an under-representation of the true value.

Analysis of MSOA demographic data using a deep neural network suggests that the observed importation rate for Bolton is not significantly lower or higher than expected. The estimated and observed total importations for each LTLA in England are shown in Figure 4. The model was able to estimate the number of imported positive cases with a good degree of accuracy, achieving an *R*^2^ score of 0.72 on the testing data and 0.86 on the training data. The model predicts that Bolton had 7.8 imports from India over the period, very close to the observed value of 7.

Using a Gradient Boosting Regression model to perform the same analysis has similar results, with the model predicting 7.1 imports over the period. The outputs of this model are shown in Figure S3 in the Supplementary Materials.

### Transmission model

The results presented above provide some indication of correlation between Indian importation rates and case rates. Further, they show that Bolton was an outlier in this relationship, indicating that the outbreak in Bolton may not have been the result of direct importation. To test this hypothesis further we must demonstrate that importations were sufficient for explaining continued transmission in some LTLA, and that the outbreak in Bolton could not be explained by importations.

**Fig. 5.**
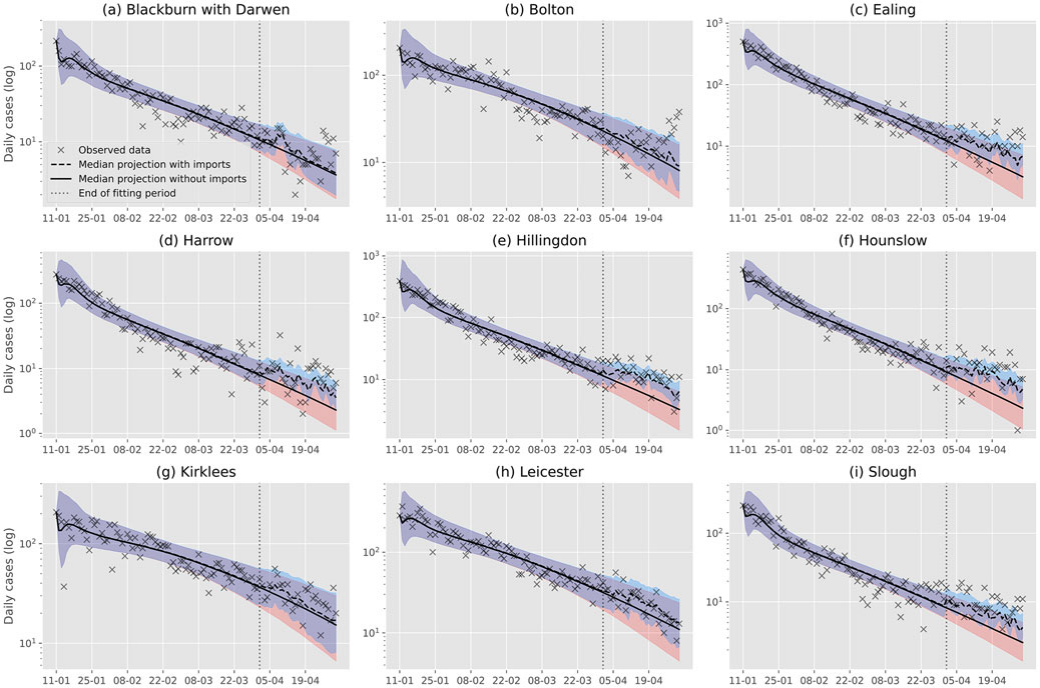
Outputs of an SEIR transmission model trained on confirmed cases between 10/01/2021 and 01/04/2021 for nine LTLAs in England. The model is run with and without importations introduced after the fit period. The 95% confidence intervals for the model with and without importations are shown in by the red and blue shaded areas, respectively. When importations are included, the model is able to predict the change in growth rates observed in Ealing, Harrow, Hillingdon, Hounslow and Slough. Increased growth rates in Bolton cannot be sufficiently explained by the importation model.

The predicted trajectories of cases with and without imports in Blackburn with Darwen, Bolton, Ealing, Harrow, Hillingdon, Hounslow, Kirklees, Leicester and Slough are shown in Figure 5. An evaluation of modelled growth rate and RMSE of log-transformed data with and without the introduction of imported cases is shown in Table 1.

**Table 1.** Observed and modelled growth rates for the model outputs shown in Figure 5, along with root mean squared error (RMSE) for the modelled data with and without importations

LTLA	Observed growth rate	Modelled growth rate without importations	Modelled growth rate with importations	RMSE without importations	RMSE with importations
Blackburn with Darwen	−0.007 (−0.035 to 0.022)	−0.019 (−0.045 to 0.006)	−0.020 (−0.044 to 0.07)	0.544	0.529
Bolton	0.006^a^ (−0.019 to 0.031)	−0.012 (−0.034 to 0.010)	−0.011 (−0.033 to 0.011)	0.588	0.559
Ealing	0.003 (−0.027 to 0.028)	−0.024 (−0.050 to 0.002)	−0.013 (−0.037 to 0.011)	0.864	0.520
Harrow	−0.002 (−0.050 to 0.046)	−0.029 (−0.081 to 0.023)	−0.016 (−0.065 to 0.033)	0.842	0.581
Hillingdon	−0.011 (−0.036 to 0.032)	−0.026 (−0.054 to 0.010)	−0.015 (−0.041 to 0.012)	0.751	0.364
Hounslow	−0.006 (−0.035 to 0.022)	−0.028 (−0.055to −0.001)	−0.014 (−0.038 to −0.011)	0.945	0.566
Kirklees	−0.006 (−0.029 to 0.017)	−0.008 (−0.029 to 0.012)	−0.009 (−0.029 to 0.012)	0.349	0.303
Leicester	−0.012 (−0.036 to 0.012)	−0.011 (−0.033 to 0.010)	−0.009 (−0.030 to 0.012)	0.326	0.261
Slough	−0.006 (−0.032 to 0.020)	−0.025 (−0.052 to 0.002)	−0.015 (−0.040 to 0.010)	0.780	0.456

a The observed values for Bolton show two distinct phases of growth. Cases decline up to 20 April with growth rate −0.002 (−0.049 to 0.046), then grow thereafter at a rate of 0.104 (0.070–0.138). The model is unable to accurately capture this change in growth rate, either with or without importations.

In Ealing, Harrow, Hillingdon, Hounslow and Slough, the observed growth rates during April were considerably higher than the base model prediction. Importations in these regions were sufficient in altering the trajectory of forecasted cases to better match that of the observed data. This indicates that the ongoing transmission occurring in these LTLAs may at least partially be explained by direct importation of positive cases.

Blackburn with Darwen, Kirklees and Leicester saw April growth rates which were similar to those predicted by the base model. Introducing importations to the model did not significantly alter these prediction trajectories. In Leicester, importations were sufficient in accounting for a brief increase in growth rate observed in early April, as well as a later return to lower growth rates.

Bolton saw increasing growth rates during the second part of April which were not accounted for by the base model. Introducing importations to the model did not result in significantly more accurate predictions. As such, it is likely that outbreak in Bolton was not a result of direct importation of positive cases.

## Discussion

This analysis has demonstrated that, over the 6-week period between 1 April and 12 May 2021, there was a positive correlation between rates of imported positive cases of COVID-19 and COVID-19 infection rates in LTLAs in England. This correlation was stronger when only considering importations from India – a country known to have significant community transmission of the Delta variant of SARS-CoV-2. This relationship persists at a finer geographic scale in many LTLAs, further demonstrating a correlative link between the two.

Bolton, however, did not follow this trend. This is perhaps surprising, as the outbreak in Bolton during the period was predominantly composed of Delta variant cases. The low rate of importation to Bolton, and the lack of a correlation between imports and local cases, indicates that the outbreak of Delta variant was not a result of direct importation. Instead, it is likely that Delta variant cases were first introduced into Bolton via secondary transmission from other LTLAs.

The causative link between importations and continued community transmission has been demonstrated in Ealing, Harrow, Hillingdon, Hounslow and Slough, where importations were shown to be a contributing factor to changes in growth rate observed in April 2021. Growth in Bolton during the second half of April, however, could not be sufficiently explained by importations. It is possible that the growth in Delta variant cases seen in Bolton was a result of secondary transmission from these LTLA, rather than direct importation from India.

While the transmission model used to investigate this causal link is appropriate for the specific task, there are some limitations which should be addressed. Throughout this paper we have relied on confirmed case data as an indicator of epidemic size. While care has been taken to account for the effects of surge testing, by eliminating any cases first identified via LFDs, there still may be inconsistencies in testing between LTLAs. Using either hospitalisation or death data in the analysis would have eliminated this source of error [20, 21], however given the slow response speeds of these data streams, the low hospitalisation and death rates over the period, and uncertainty over the effect of vaccination on these outcomes; it was, on balance, more appropriate to use case data in this instance.

The transmission model presented is relatively simple, and should be regarded as an analysis tool, rather than a forecasting tool. The lack of age structure in the model is a significant source of error, particularly with regard to the effect of vaccination. Without age structure, we assume that all susceptible individuals have the same contribution to the rate of infection. This is not true, and in many cases the age groups with the highest rates of transmission are those with the lowest vaccination rates [22]. However, as case numbers were low over the period of this study, further stratification of cases would have had a significant impact on the ability to fit the model.

The model does not account for any changes in transmission rate which may have occurred over the period and does not allow for any movement of individuals between LTLAs. The model also treats importations as exerting the same transmissive pressure as native cases. This fails to accurately represent relative differences in transmissibility between variants [23, 24] as well differences in contact network structures between imported and native cases. A more sophisticated model which captures these nuances may be able to accurately quantify the extent to which importations influenced growth rates in these communities; however, this was not a necessary requirement for this analysis, and would not have been supported in a low data environment. Rather, the ability to act quickly on readily available data streams with simple but effective techniques was crucial in providing rapid and valuable analysis.

Despite the limitations of the model, there is good evidence that importations played an important role in the ongoing community transmission observed in a number of LTLAs – and further, that the growth rates observed in Bolton were not sufficiently explained by importations. From this we can conclude that the likely source of the outbreak of Delta variant in Bolton was due to secondary transmission from cases in those LTLAs where importations were a significant contributing factor to community transmission.

The results presented in this paper have highlighted the importance of border screening in providing a fast and effective way to monitor imported cases of COVID-19. As new variants of SARS-CoV-2 emerge around the world, there will be an ongoing need to provide rapid assessment of traveller origins and destinations – and to identify possible outbreaks caused by incursion of new variants. While genetic sequencing of cases is vital in helping us identify and monitor new variants, the techniques presented here provide a way to rapidly respond to developing situations. Further, the techniques are able to identify likely inter-region transmission of new variants, and can provide an early warning system for their establishment.

The results of this analysis present an important question regarding the spread of the Delta variant in April 2021 – why did we see the variant enter epidemic phase in Bolton, while LTLAs with high rates of importation from India, such as Hounslow, Hillingdon and Leicester, did not see such quick growth? Answering this question will require and in-depth epidemiological study of the factors influencing the establishment of Delta variant in Bolton. Such a study would be deeply valuable in helping us understand how new variants become established in the UK, and will help us identify regions which might be prone to similar outbreaks in the future.

## Data Availability

This paper relies on individually identifiable data collected by Public Health England surveillance streams. Unfortunately, it is not possible to make these data available to the public. The code used to process the data is available on request.

## Supplementary material

For supplementary material accompanying this paper visit https://doi.org/10.1017/S0950268821002776. click here to view supplementary material
