# Supplementary material for: Assessing the role of imported cases on the establishment of SARS-CoV-2 Delta variant of concern in Bolton, UK
Source: Epidemiol Infect. 2022 May 12;150:e100. doi: 10.1017/S0950268821002776 (PMC9151661; doi:10.1017/S0950268821002776)
Supplement: Supplementary file 1 [file S0950268821002776sup001.docx]

# SUPLEMENTARY MATERIAL

## S1 Machine learning models for estimating importations

The neural network used to estimate imported cases to MSOA in England consists of an input layer, $L_{\mathrm{in}}\in\mathbb{R}^{35}$, followed by three hidden layers; $H_{1}\in\mathbb{R}^{16}$, $H_{2}\in\mathbb{R}^{16}$, $H_{3}\in\mathbb{R}^{4}$, and an output layer $L_{out}\in\mathbb{R}^{1}$. The input layer and three hidden layers have dropout applied with probability $p=0.2$ and Reticulated Linear Unit activation functions. The Output layer has no dropout and uses a linear activation function. The model is compiled with Mean Squared Error loss and uses the Adam optimizer.

Importations were also estimated using a Gradient Boosting Regressor machine learning algorithm, giving similar results to the Neural Network. The regressor uses 5000 estimators, with a maximum depth of 4 and learning rate 0.001; and monitors loss using the least-squares algorithm. A plot of estimated vs observed importations to LTLA using this model is shown in figure S1.


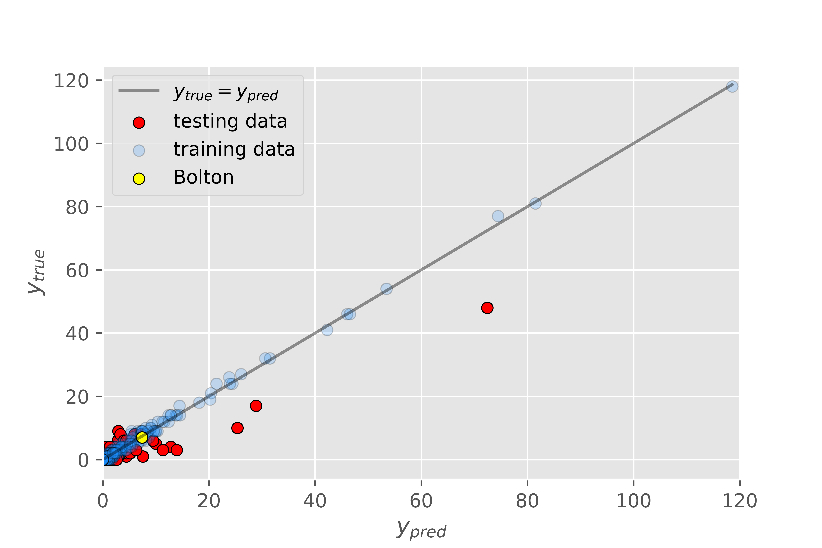


Figure S1: Observed vs predicted importations to LTLA in England using a Gradient Boosting Regressor algorithm. The algorithm predicts 7.4 importations for Bolton between 1st of April and 12th of May 2021, close to the observed value of 7.

## S2 MSOA importations from Pakistan and from all countries

Figure S2 shows the relationship between MSOA case rate per 1000 population and importation rate from Pakistan for the nine LTLA discussed in the main body of the paper. The analysis suggests that the correlation between case rates and importations from Pakistan is weaker in Blackburn with Darwen, Leicester, Ealing and Hounslow; while Harrow and Slough both maintain the same weak correlations. Bolton and Hillingdon both have strong correlations, although these are primarily driven by single MSOA with high importations and high case rates. Statistical significance is low when these MSOA are excluded from the analysis ($p=0.143$ and $p=0.09$ respectively). Kirklees has a stronger relationship when considering Pakistani imports, and this relationship is robust to the removal of the highest scoring MSOA.


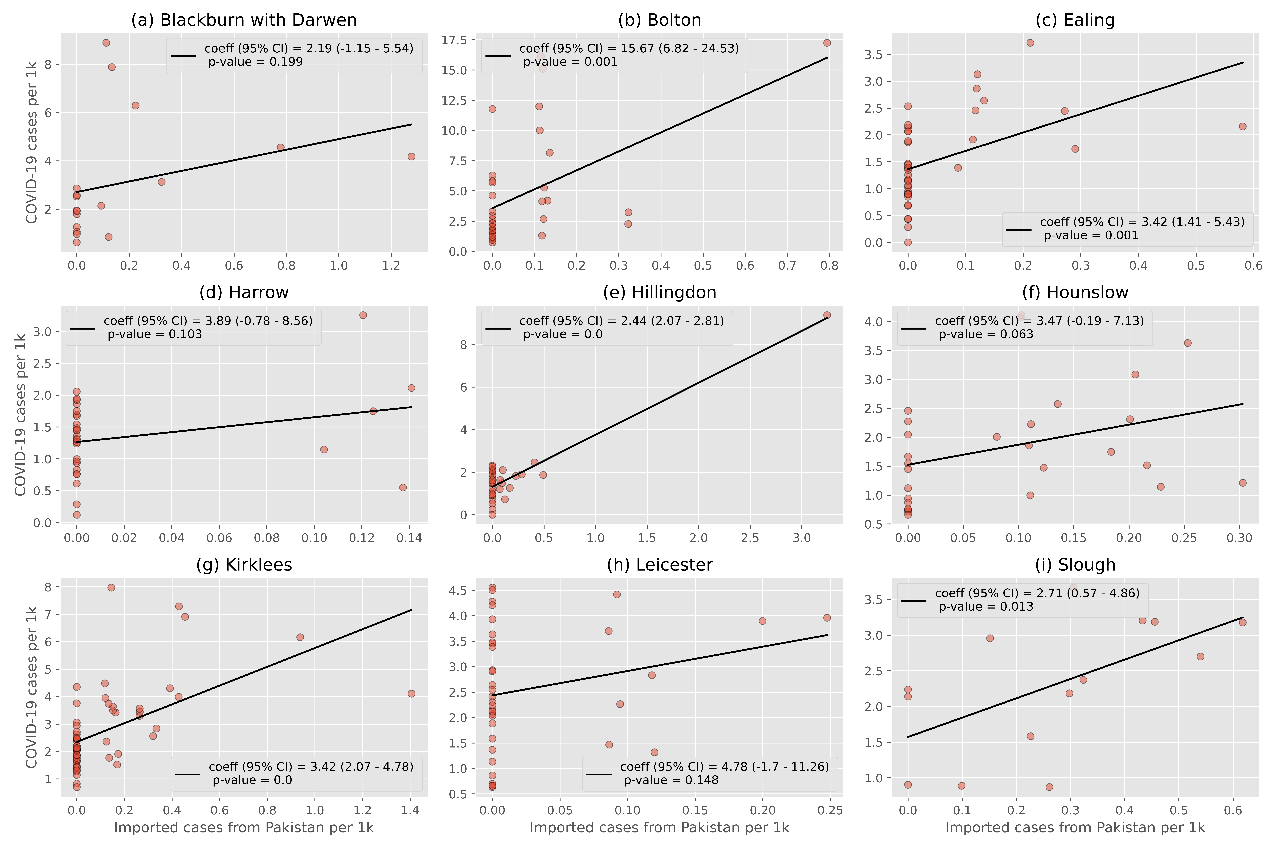
Analysis of importations from all countries, shown in figure S3, suggests a stronger relationship with MSOA case rates than for Pakistan or India alone. However, the relationship in Bolton is again driven by a single MSOA with high importation rates and high case rates. When this MSOA is removed the relationship becomes considerably less statistically significant ($p=0.042$).

Figure S2: MSOA importation rate per 1k population vs COVID-19 case rate per 1k popultion for the nine LTLA discussed in the main body of the paper. Most LTLA remain broadly unchanged when considering importations from Pakistan alone. Bolton and Hilingdon both show statistically significant correlations, although in each case these are driven by a single MSOA with high importation and case rates. The strong correlation observe din Kirklees, however, is robust to the removal of specific MSOA.


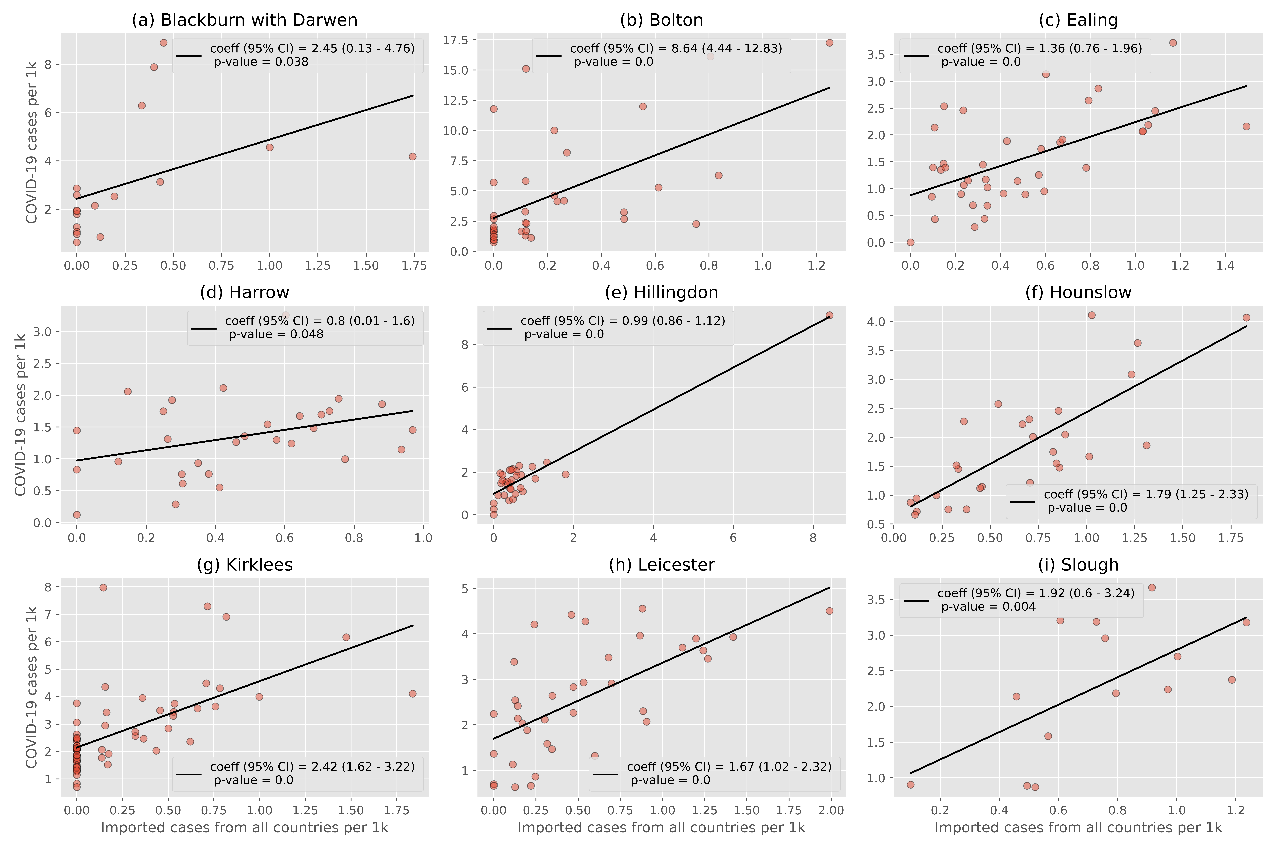


Figure S3: Importation rate from all countries per 1k population vs case rate per 1k population for MSOA in the nine LTLA discussed in the main body of the paper. Blackburn with Darwen is the LTLA which sees reduced statistical significance compared to importations form India alone. The relationship for Bolton is again driven by a single MSOA with high importation rates and high overall case numbers, removal of this MSOA results in reduced statistical significance ($p=0.042$)

## S3 Model parameters and initial values

Table S1 provides details on the parameters used in the Transmission model discussed in the main body of this paper. Priors have been included for parameters which were identified through ABC fitting. Initial state values are shown in table S2, once again priors have been provided for initial states that were estimated via ABC fitting.

| Parameter | Interpretation | Value | ABC Prior |
| --- | --- | --- | --- |
| $\boldsymbol{N}_{\boldsymbol{0}}$ | Population | Various, obtained from ONS population estimates | N/A |
| $\boldsymbol{\beta}$ | Transmission Rate | Inferred in ABC fitting | - 1. $\leq\beta\leq1$ |
| $\boldsymbol{\alpha}$ | Rate of transition through latent period | $1/5.2 days^{-1}$ [9, 10] | N/A |
| $\boldsymbol{k}$ | Proportion of infectious individuals who are detected | Inferred in ABC fitting | $0 \leq k \leq1$ |
| $\boldsymbol{\gamma}$ | Rate of transition through infectious period | Inferred in ABC fitting | - 1. $\leq\gamma\leq1$ |
| $\boldsymbol{\delta}_{\boldsymbol{d}}$ | Rate of removal of detected infectious individuals | Inferred in ABC fitting | $0.05\leq\delta_{d}\leq1$ |
| $\boldsymbol{\delta}_{\boldsymbol{u}}$ | Rate of removal of undetected infectious individuals | Inferred in ABC fitting | $0.05\leq\delta_{u}\leq1$ |
| $\boldsymbol{\phi}$ | Vaccination rate | Various, obtained from PHE records. [1] | N/A |
| $\boldsymbol{\nu}$ | Reduction in transability of detected infectious individuals | 0.1 [11] | N/A |

Table S1: Parameters used in the transmission model.

| State | Initial value | Priors |
| --- | --- | --- |
| $\boldsymbol{S}_{\boldsymbol{0}}$ | Inferred in ABC fitting | $\frac{N_{0}}{3}\leq S_{0}\leq\frac{9N_{0}}{10}$ |
| $\boldsymbol{E}_{\boldsymbol{0}}$ | Inferred in ABC fitting | $1\leq E_{0}\leq{10}^{4}$ |
| $\boldsymbol{E}_{\boldsymbol{1}}$ | Inferred in ABC fitting | $1\leq E_{1}\leq{10}^{4}$ |
| $\boldsymbol{E}_{\boldsymbol{2}}$ | Inferred in ABC fitting | $1\leq E_{2}\leq{10}^{4}$ |
| $\boldsymbol{I}_{\boldsymbol{0}}$ | Inferred in ABC fitting | $1\leq I_{0}\leq{10}^{4}$ |
| $\boldsymbol{I}_{\boldsymbol{d}}$ | $9y(0)$ | N/A |
| $\boldsymbol{I}_{\boldsymbol{u}}$ | Inferred in ABC fitting | $1\leq I_{u}\leq{10}^{4}$ |
| $\boldsymbol{R}$ | Inferred in ABC fitting | $\frac{N_{0}}{10}\leq S_{0}\leq\frac{2N_{0}}{3}$ |

Table S2: Initial states used in the transmission model. The initial value of $I_{d}$ is given as $I_{d}\left( 0 \right)=T_{c}y\left( 0 \right)$where $T_{c}$ is the mean infectious period, estimated as 9 days, and $y(0)$ is the first observed daily incidence. Sensitivity to the value of $T_{c}$ is discussed in section S4.

## S4 Parameter sensitivity

Table S3 shows how central estimates of growth rate and RMSE vary with vaccine efficacy, $\phi$, across each LTLA. A box plot of central estimates of growth rate across three values of $\phi$ is shown in figure S4. As vaccine efficacy is increased, the estimated growth rates decrease. Estimated growth rates are higher when importations are included in the model, however there is no indication that this relationship is significantly affected by changes in vaccine efficacy.

Sensitivity of the model to changes in the relative transmissibility of an infectious individual upon detection,$\nu$, is shown in table S4, with a box plot of the central predictions of growth rates with and without importations at each value of $\nu$ shown in figure S5. As $\nu$ increases, and so isolation effectiveness falls, we see a gradual increase in growth rates. This increase is slightly more pronounced when the model includes importations.

Table S5 shows the sensitivity of the model to the value of the mean generation time, $T_{c}$. Rather than acting as an explicit parameter in the model, $T_{c}$ scales the initial detect population according to $I_{d}\left( 0 \right)=T_{c}y\left( 0 \right)$. Changes in $T_{c}$ have little to no effect on the estimate growth rates in the model after the fit period, both with and without importations.

| $\boldsymbol{\phi= 0.4}$ | | | | |
| --- | --- | --- | --- | --- |
| LTLA | **Modelled growth rate without imports (central estimate)** | **Modelled growth rate with imports (central estimate)** | **RMSE without imports** | **RMSE with imports** |
| Blackburn with Darwen | -0.0167 | -0.0184 | 0.512 | 0.515 |
| Bolton | -0.0108 | -0.0095 | 0.551 | 0.539 |
| Ealing | -0.0224 | -0.012 | 0.799 | 0.498 |
| Harrow | -0.0255 | -0.0146 | 0.819 | 0.570 |
| Hillingdon | -0.0226 | -0.0125 | 0.747 | 0.367 |
| Hounslow | -0.0258 | -0.0130 | 0.880 | 0.551 |
| Kirklees | -0.0071 | -0.0075 | 0.286 | 0.283 |
| Leicester | -0.0106 | -0.0087 | 0.300 | 0.284 |
| Slough | -0.0229 | -0.0137 | 0.732 | 0.426 |
| $\boldsymbol{\phi= 0.6}$ | | | | |
| Blackburn with Darwen | -0.0173 | -0.019 | 0.518 | 0.517 |
| Bolton | -0.0121 | -0.106 | 0.578 | 0.556 |
| Ealing | -0.0243 | -0.0128 | 0.849 | 0.516 |
| Harrow | -0.0270 | -0.0154 | 0.847 | 0.577 |
| Hillingdon | -0.0218 | -0.0123 | 0.711 | 0.358 |
| Hounslow | -0.0280 | -0.0135 | 0.927 | 0.558 |
| Kirklees | -0.0083 | -0.0086 | 0.322 | 0.288 |
| Leicester | -0.0114 | -0.0092 | 0.325 | 0.264 |
| Slough | -0.0240 | -0.0142 | 0.765 | 0.446 |
| $\boldsymbol{\phi= 0.9}$ | | | | |
| Blackburn with Darwen | -0.0194 | -0.0208 | 0.541 | 0.529 |
| Bolton | -0.0139 | -0.0121 | 0.629 | 0.584 |
| Ealing | -0.0246 | -0.0130 | 0.848 | 0.518 |
| Harrow | -0.0292 | -0.0161 | 0.875 | 0.585 |
| Hillingdon | -0.0249 | -0.0133 | 0.788 | 0.374 |
| Hounslow | -0.0300 | -0.0144 | 0.964 | 0.568 |
| Kirklees | -0.0100 | -0.0097 | 0.376 | 0.321 |
| Leicester | -0.0124 | -0.0102 | 0.347 | 0.252 |
| Slough | -0.0281 | -0.0158 | 0.842 | 0.486 |


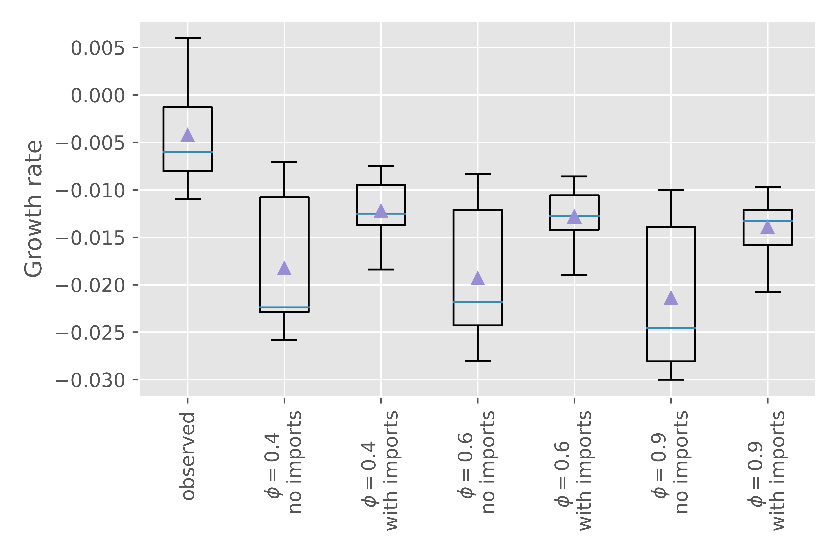
Table S3: Sensitivity of the results to changes in the vaccine efficacy, $\phi$.

Figue S4: Boxplot showing how central estimate for growth rate varies with vaccine efficacy, 𝜙.

| $\boldsymbol{\nu= 0.05}$ | | | | |
| --- | --- | --- | --- | --- |
| LTLA | **Modelled growth rate without imports** | **Modelled growth rate with imports** | **RMSE without imports** | **RMSE with imports** |
| Blackburn with Darwen | -0.0191 | -0.0207 | 0.535 | 0.527 |
| Bolton | -0.0131 | -0.0115 | 0.608 | 0.571 |
| Ealing | -0.0252 | -0.0134 | 0.862 | 0.526 |
| Harrow | -0.0269 | -0.0158 | 0.837 | 0.580 |
| Hillingdon | -0.0262 | -0.0141 | 0.829 | 0.389 |
| Hounslow | -0.0301 | -0.0144 | 0.967 | 0.569 |
| Kirklees | -0.0094 | -0.0097 | 0.356 | 0.310 |
| Leicester | -0.0119 | -0.0100 | 0.334 | 0.255 |
| Slough | -0.0262 | -0.0154 | 0.800 | 0.466 |
| $\boldsymbol{\nu= 0.2}$ | | | | |
| Blackburn with Darwen | -0.0195 | -0.0210 | 0.546 | 0.529 |
| Bolton | -0.0132 | -0.0113 | 0.607 | 0.568 |
| Ealing | -0.0241 | -0.0123 | 0.842 | 0.508 |
| Harrow | -0.0290 | -0.0155 | 0.869 | 0.579 |
| Hillingdon | -0.0243 | -0.0123 | 0.778 | 0.367 |
| Hounslow | -0.0313 | -0.0134 | 1.002 | 0.568 |
| Kirklees | -0.0096 | -0.0096 | 0.373 | 0.314 |
| Leicester | -0.0119 | -0.0095 | 0.343 | 0.260 |
| Slough | -0.0270 | -0.0149 | 0.823 | 0.468 |
| $\boldsymbol{\nu= 0.5}$ | | | | |
| Blackburn with Darwen | -0.0204 | -0.0211 | 0.544 | 0.524 |
| Bolton | -0.013 | -0.0109 | 0.607 | 0.560 |
| Ealing | -0.0236 | -0.0115 | 0.827 | 0.493 |
| Harrow | -0.0287 | -0.0144 | 0.872 | 0.571 |
| Hillingdon | -0.0251 | -0.0116 | 0.816 | 0.372 |
| Hounslow | -0.0296 | -0.0126 | 0.962 | 0.558 |
| Kirklees | -0.0091 | -0.0089 | 0.376 | 0.321 |
| Leicester | -0.0117 | -0.0088 | 0.337 | 0.227 |
| Slough | -0.0264 | -0.0138 | 0.816 | 0.449 |

Table S4: Sensitivity of results to changes in relative transmissibility of detected infectious individuals, $\nu$.


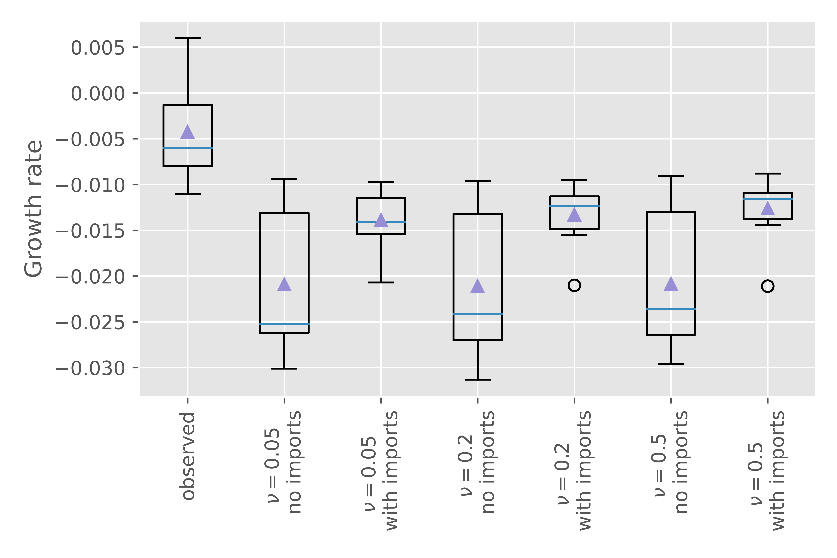


Figure S5: Boxplot describing how central estimate for growth rate varies with relative transmissibility of detected infectious individuals, $\nu$.

| $\boldsymbol{T}_{\boldsymbol{c}}\boldsymbol{=3 days}$ | | | | |
| --- | --- | --- | --- | --- |
| LTLA | **Modelled growth rate without imports** | **Modelled growth rate with imports** | **RMSE without imports** | **RMSE with imports** |
| Blackburn with Darwen | -0.0191 | -0.0208 | 0.536 | 0.526 |
| Bolton | -0.0129 | -0.0113 | 0.599 | 0.568 |
| Ealing | -0.025 | -0.0129 | 0.872 | 0.523 |
| Harrow | -0.0281 | -0.0156 | 0.849 | 0.580 |
| Hillingdon | -0.0246 | -0.0131 | 0.796 | 0.377 |
| Hounslow | -0.0292 | -0.0138 | 0.940 | 0.560 |
| Kirklees | -0.0095 | -0.0098 | 0.373 | 0.320 |
| Leicester | -0.0120 | -0.0098 | 0.330 | 0.258 |
| Slough | -0.0261 | -0.015 | 0.810 | 0.469 |
| $\boldsymbol{T}_{\boldsymbol{c}}\boldsymbol{=6 days}$ | | | | |
| Blackburn with Darwen | -0.0189 | -0.0203 | 0.533 | 0.525 |
| Bolton | -0.0131 | -0.0114 | 0.607 | 0.570 |
| Ealing | -0.0239 | -0.0128 | 0.836 | 0.514 |
| Harrow | -0.0291 | -0.0160 | 0.871 | 0.583 |
| Hillingdon | -0.0258 | -0.0135 | 0.836 | 0.388 |
| Hounslow | -0.0304 | -0.0143 | 0.959 | 0.565 |
| Kirklees | -0.0091 | -0.0094 | 0.349 | 0.304 |
| Leicester | -0.0123 | -0.0099 | 0.356 | 0.255 |
| Slough | -0.0247 | -0.0147 | 0.766 | 0.447 |
| $\boldsymbol{T}_{\boldsymbol{c}}\boldsymbol{=12 days}$ | | | | |
| Blackburn with Darwen | -0.0188 | -0.0203 | 0.532 | 0.524 |
| Bolton | -0.0138 | -0.012 | 0.625 | 0.581 |
| Ealing | -0.0267 | -0.0135 | 0.915 | 0.539 |
| Harrow | -0.0303 | -0.0164 | 0.896 | 0.589 |
| Hillingdon | -0.0267 | -0.0136 | 0.855 | 0.392 |
| Hounslow | -0.0321 | -0.0145 | 1.002 | 0.574 |
| Kirklees | -0.0094 | -0.0096 | 0.361 | 0.310 |
| Leicester | -0.0125 | -0.0100 | 0.349 | 0.254 |
| Slough | -0.0256 | -0.0149 | 0.800 | 0.464 |
| $\boldsymbol{T}_{\boldsymbol{c}}\boldsymbol{=15 days}$ | | | | |
| Blackburn with Darwen | -0.0191 | -0.0209 | 0.540 | 0.527 |
| Bolton | -0.0129 | -0.0113 | 0.599 | 0.568 |
| Ealing | -0.0253 | -0.0132 | 0.874 | 0.526 |
| Harrow | -0.0287 | -0.0161 | 0.873 | 0.585 |
| Hillingdon | -0.0256 | -0.0135 | 0.817 | 0.383 |
| Hounslow | -0.0313 | -0.0142 | 0.995 | 0.572 |
| Kirklees | -0.0093 | -0.0097 | 0.365 | 0.314 |
| Leicester | -0.0119 | -0.0098 | 0.337 | 0.257 |
| Slough | -0.0271 | -0.0152 | 0.834 | 0.479 |

Table S4: Sensitivity of results to changes in mean generation time, $T_{c}$.


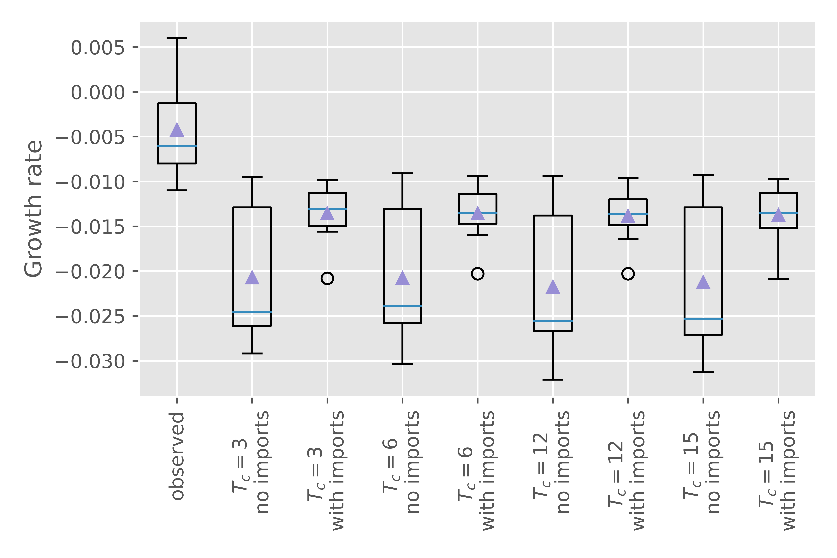


Figure S6: Boxplot describing how central estimate for growth rate varies with mean generation time, $T_{c}$.
